# Hemosiderin-laden macrophages are an independent factor correlated with pulmonary vascular resistance in idiopathic pulmonary fibrosis: a case control study

**DOI:** 10.1186/s12890-017-0376-8

**Published:** 2017-02-06

**Authors:** Jun Fukihara, Hiroyuki Taniguchi, Masahiko Ando, Yasuhiro Kondoh, Tomoki Kimura, Kensuke Kataoka, Taiki Furukawa, Takeshi Johkoh, Junya Fukuoka, Koji Sakamoto, Yoshinori Hasegawa

**Affiliations:** 1 Department of Respiratory Medicine and Allergy, Tosei General Hospital, 160 Nishioiwake-cho, Seto-shi, Aichi 489-8642 Japan; 2 Center of Advanced Medicine and Clinical Research, Nagoya University Hospital, 65 Tsurumai-cho, Showa-ku, Nagoya-shi, Aichi 466-0065 Japan; 3 Department of Radiology, Kinki Central Hospital of Mutual Aid Association of Public School Teachers, 3-1 Kurumazuka, Itami-shi, Hyogo 664-8533 Japan; 4 Department of Pathology, Nagasaki University Graduate School of Biomedical Sciences, 1-12-4 Sakamoto, Nagasaki-shi, Nagasaki Japan; 5 Department of Respiratory Medicine, Nagoya University Graduate School of Medicine, 65 Tsurumai-cho, Showa-ku, Nagoya-shi, Aichi 466-0065 Japan

**Keywords:** Hemosiderin-laden macrophages, Idiopathic pulmonary fibrosis, Pulmonary vascular resistance, Mean pulmonary arterial pressure, Bronchoalveolar-lavage, Right heart catheterization

## Abstract

**Background:**

Increases in hemosiderin-laden macrophages (HLM) are reported to be observed in idiopathic pulmonary fibrosis (IPF). According to a recent study, significant correlation between hemosiderin deposition in the lung tissue of IPF and pulmonary hypertension evaluated by echocardiography has been suspected. In this study, we aimed to evaluate whether HLM in bronchoalveolar lavage fluid (BALF) is a factor correlated with pulmonary hemodynamic parameters evaluated by right heart catheterization in patients with IPF.

**Methods:**

Initial data from 103 consecutive patients with IPF who underwent surgical lung biopsy between November 2007 and March 2014 were retrospectively analyzed. The “HLM score” of BALF was established by dividing the number of Perls’ Prussian blue stain positive macrophages by the total number of macrophages counted.

**Results:**

BALF showed an elevated HLM score (38.2%). Right heart catheterization revealed mean pulmonary arterial pressure (mPAP) of 16.3 mmHg and pulmonary vascular resistance (PVR) of 1.55 Wood units. HLM score was positively correlated with mPAP (ρ = 0.204; *p* = 0.038) and PVR (ρ = 0.349, *p* < 0.001). In multivariate analysis, 6-min walk distance (standardized partial regression coefficient [β], −0.391; *p* < 0.001), minimum oxygen saturation during 6-min walk distance (β, −0.294; *p* = 0.001) and HLM score (β, 0.265; *p* = 0.002) were independently correlated with PVR.

**Conclusions:**

HLM score in BALF is an independent factor correlated with PVR in patients with IPF.

## Background

Hemosiderin-laden macrophages (HLM) in bronchoalveolar lavage fluid (BALF) were originally known as a diagnostic biomarker of alveolar hemorrhage [1, 2]. Recently, hemosiderin deposition or exaggerated numbers of HLM have also been observed in idiopathic pulmonary fibrosis (IPF) [3, 4], which is a progressive interstitial lung disease characterized by a poor prognosis, limited response to treatment and a histopathological pattern of usual interstitial pneumonia (UIP) [5]. In these reports, a relationship between hemosiderin deposition and elevated pulmonary arterial pressure was suspected [3, 4].

In 2010, Kim et al. reported that iron deposition in the lung tissue of IPF is significantly correlated with elevated right ventricular systolic pressure (RVSP) measured by echocardiography [3]. Puxeddu et al. later demonstrated elevated HLM in the BALF of patients with IPF, especially of severer patients and patients with higher RVSP [4]. However, whether HLM in the BALF of patients with IPF is correlated with parameters measured by right heart catheterization (RHC) has never been clarified.

Among parameters measured by RHC, several recent studies have reported that pulmonary vascular resistance (PVR) is the strongest prognostic factor of interstitial lung diseases [6, 7]. While there are some reports on predictive factors for mean pulmonary arterial pressure (mPAP) [8–12], such as diffusing capacity of the lung for carbon monoxide (D_L_CO) [8–10], 6-min walk distance (6MWD) [11, 12], minimum oxygen saturation during 6-min walk test (min-SpO_2_) [9, 11] and others, the correlation between PVR and other clinical variables has not been well discussed.

The aim of this retrospective study was to evaluate whether HLM in BALF is correlated with PVR in patients with IPF, together with other possible correlating factors measured at initial evaluation, such as pulmonary function, oxygen saturation and 6-min walk test measurements.

## Methods

### Study population

**Fig. 1:**
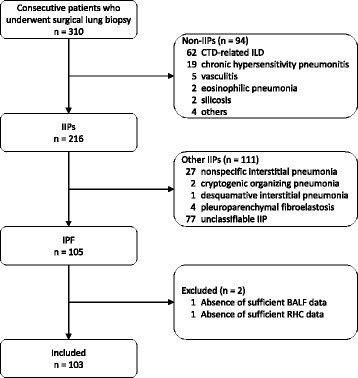
Screening and inclusion process for patients in the study. Definition of abbreviations: *IIP* idiopathic interstitial pneumonia, *CTD* connective tissue disease, *ILD* interstitial lung disease, *IPF* idiopathic pulmonary fibrosis, *BALF* bronchoalveolar lavage fluid, *RHC* right heart catheterization

This study was approved by Institutional Review Board of Tosei General Hospital (Review Board No. 480, Seto, Aichi, Japan). Informed consent has not been obtained from participants because this is a retrospective study and the data were analyzed anonymously. A retrospective review of the initial evaluation data from 310 consecutive patients with interstitial lung disease who underwent surgical lung biopsy at Tosei General Hospital between November 2007 and March 2014 was undertaken. After excluding 94 cases with connective tissue diseases (patients fulfilling the American College of Rheumatology criteria for rheumatoid arthritis [13], systemic lupus erythematosus [14, 15], systemic sclerosis [16], Sjögren’s syndrome [17], polymyositis-dermatomyositis [18], or mixed connective tissue disease [19]), hypersensitivity pneumonitis, vasculitis or other known causes, 216 patients were diagnosed with idiopathic interstitial pneumonia. From 2011 onward, multidisciplinary diagnoses were made after close communication between clinicians, radiologists and pathologists, and 105 patients with IPF were detected according to the guidelines for IPF [5]. After excluding one patient whom HLM in BALF could not be evaluated due to insufficient total cell count and one who did not take RHC, a total of 103 patients were finally enrolled in this study (Fig. 1). Pulmonary arterial wedge pressure of all of 103 patients was ≤ 15 mmHg. After conducting a clinical workup, we performed bronchoalveolar lavage (BAL) and RHC as a part of the initial evaluation before surgical lung biopsy. Characteristics of patients before BAL, including demographics, detailed clinical history, results of pulmonary function tests, serologic tests, arterial blood gas analysis and six-minute walk tests, were collected from their clinical charts.

### Pulmonary function tests

Pulmonary function tests performed before BAL (within 1 month) were used for analysis. Forced vital capacity and D_L_CO were measured by means of CHESTAC-55 V (Chest, Tokyo, Japan). Pulmonary function was measured according to the American Thoracic Society/European Respiratory Society recommendation as a physiological assessment [20, 21]. The results were expressed as percentages of the normal predicted values.

### High resolution computed tomography

High resolution computed tomography was performed with 0.5-mm thick sections. An expert thoracic radiologist with 26 years of experience who was blinded to the clinical information or histological diagnosis reviewed the scans. The probability of usual interstitial pneumonia was evaluated and categorized according to the guidelines for IPF [5].

### Six-minute walk test

Six-minute walk test was conducted according to the American Thoracic Society statement [22]. Briefly, all patients were tested under standardized conditions by trained technicians. Patients were instructed to walk as far as possible in 6 min. The distance that patients could walk was recorded as 6MWD. Oxygen saturation was also measured by pulse oximetry during the test and min-SpO_2_ was recorded. Patients underwent the tests twice to minimize the training effects.

### Bronchoalveolar lavage and detection of hemosiderin laden macrophages

Flexible bronchoscopy was performed and processed following a method described in the literature [23]. A flexible bronchoscope (BF-1 T260; Olympus Optical Co., Tokyo, Japan) was wedged into a segmental bronchus of the middle lobe or the lingula. Sequential infusions of 50 mL sterile normal saline were instilled three times, then immediately aspirated by manual suction after each instillation through the bronchoscope. The total volume of recovered fluid was measured, and a sample of the fluid was used for bacteriological and fungal studies. The remaining fluid was filtered through a double layer of surgical gauze and used for cell counts and cytological examination.

The cell pellets were separated from supernatant by low-speed centrifugation at 300 × g for 5 min at 4 °C (CytoSpin; Thermo Fischer Scientific Inc., Waltham, MA). Differential cell counts were made from a total count of ≥ 300 cells stained by Diff-Quick™ stain (Scientific Products, McGraw Park, IL). HLM were detected by Perls’ Prussian blue stain and counted according to the procedure of De Lassence et al. [24]. Two hundred alveolar macrophages were examined at a magnification of × 500. The “HLM score” was established by dividing the number of Perls’ Prussian blue stain-positive macrophages by the total number of macrophages counted.

### Right heart catheterization

RHC was performed percutaneously using a Swan-Ganz catheter via either the cubital vein or the femoral vein. Cardiac output and cardiac index were calculated by the thermodilution method. PVR was calculated using the formula: PVR = (mPAP – pulmonary arterial wedge pressure)/cardiac output.

### Surgical lung biopsy and pathological diagnosis

Surgical lung biopsies were performed by either open lung biopsy or video-assisted thoracoscopic surgery. Formalin-fixed, paraffin-embedded tissue was used for this study. For proof of histology, all available lung biopsy specimens were reviewed by a pulmonary pathologist with 20 years of experience who was blinded to clinical and radiological information.

### Statistical analysis

Continuous variables were presented as mean ± standard deviation or median (range), as appropriate. Categorical variables were summarized by frequency and percentage.

Univariate relationships between mPAP or PVR and other variables were evaluated using Spearman rank correlation test for continuous variables or Student’s *t* test for categorical variables, since PVR had a normal distribution (data not shown). To avoid multicollinearity, only one of the highly correlated variables (correlation coefficient ≥ 0.7) was entered in the multivariate model predicting PVR, if present. The stepwise linear regression analysis model was constructed to identify independent predictors of PVR. All statistical tests were two sided, and values of < 0.05 were considered statistically significant. Statistical analyses were carried out using SPSS version 21.0 (SPSS Inc., Chicago, IL).

## Results

### Patient characteristics

**Fig. 2:**
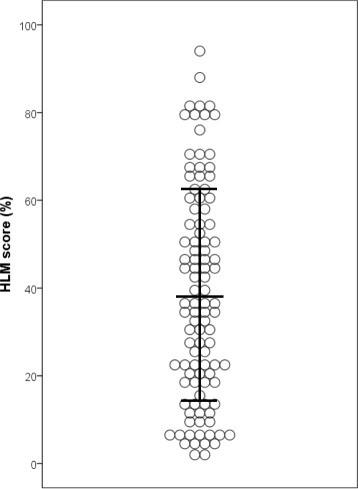
Scatter plot of HLM score. Data is shown as mean ± SD. Definition of abbreviation: *HLM* hemosiderin-laden macrophages

All the initial evaluation exams, including pulmonary function test, high resolution computed tomography, six-minute walk test, BAL and RHC, of the 103 patients enrolled in this study were performed in a median time of 22 days (range 2–115). Their clinical characteristics (Table 1) showed a predominance of males and patients with smoking history. Median age was 66 years old. Baseline predicted value for forced vital capacity and D_L_CO were 86.8 ± 20.3% and 63.7 ± 20.3% of normal predicted value, respectively. Partial pressure of oxygen in arterial blood was 82.4 ± 11.4 Torr in room air. 6MWD was 608 ± 140 m with min-SpO_2_ of 85.0 ± 7.6%. BALF showed almost normal differential cell counts with elevated HLM score (38.2 ± 24.2%, Table 2, Fig. 2). RHC revealed mPAP of 16.3 ± 4.2 mmHg and PVR of 1.55 ± 0.81 Wood units with normal left heart function (Table 3).

**Table 1:** Characteristics of patients before bronchoalveolar lavage

Characteristics	*N* = 103
Age, years	66 (48 ~ 76)
Male gender	80 (77.7)
Smokers	80 (77.7)
Brinkman index	630 ± 581
BNP, pg/ml	28.7 ± 38.7
Pulmonary function test	
FVC, % pred	86.8 ± 20.3
D_L_CO, % pred	63.7 ± 20.3
Arterial blood gas analysis	
PaO_2_, Torr	82.4 ± 11.4
PaCO_2_, Torr	41.0 ± 3.6
Six-minute walk test	
Six-minute walk distance, m	608 ± 140
Min-SpO_2_, %	85.0 ± 7.6

Values are presented as number (%), mean ± standard deviation or median (range). Definitions of abbreviations: *BNP* brain natriuretic peptide, *FVC* forced vital capacity, *% pred* percent of normal predicted value, *D*_*L*_ *CO* diffusing capacity of the lung for carbon monoxide, *PaO*_*2*_ partial pressure of oxygen in arterial blood, *PaCO*_*2*_ partial pressure of carbon dioxide in arterial blood, *min-SpO*_*2*_ minimum oxygen saturation during 6-min walk test

**Table 2:** Bronchoalveolar lavage fluid analysis

Characteristics	*N* = 103
Total cell counts, ×10^5^/ml	1.98 ± 1.87
Differential cell count	
Neutrophils, %	0.3 (0.0 ~ 63.6)
Lymphocytes, %	2.5 (0.0 ~ 36.8)
Eosinophils, %	0.2 (0.0 ~ 35.0)
Macrophages, %	94.0 (34.8 ~ 99.8)
HLM score, %	38.2 ± 24.2

Values are presented as mean ± standard deviation or median (range). Definition of abbreviation: *HLM* hemosiderin-laden macrophage

**Table 3:** Pulmonary arterial catheterization analysis

Characteristics	*N* = 103
mPAP, mmHg	16.3 ± 4.2
PVR, Wood units	1.55 ± 0.81
PVRI, Wood units × m^2^	2.66 ± 1.32
PAWP, mmHg	7.8 ± 3.3
CI, L/min/m^2^	3.1 ± 0.5

Values are presented as mean ± standard deviation. Definitions of abbreviations: *mPAP* mean pulmonary arterial pressure, *PVR* pulmonary vascular resistance, *PVRI* pulmonary vascular resistance index, *PAWP* pulmonary arterial wedge pressure, *CI* cardiac index

### Correlation between mPAP, PVR and HLM scores

HLM score showed significant positive correlations with mPAP (ρ = 0.204; *p* = 0.038) and PVR (ρ = 0.349, *p* < 0.001). Due to the clinical significance of PVR compared with mPAP as a prognostic factor for interstitial lung diseases [6, 7], PVR was selected for use in the following analysis in this study.

### Factors correlated with PVR

In the univariate analysis, partial pressure of oxygen in arterial blood, D_L_CO, 6MWD and min-SpO_2_ were negatively correlated with PVR, while age and HLM score were positively correlated (Table 4). No significant multicollinearity was found between the variables shown in table 4. In the stepwise linear regression analysis including all of the variables shown in table 4, 6MWD (standardized partial regression coefficient [β], −0.391; 95% confidence interval [95% CI], −0.587 ~ −0.196; *p* < 0.001), min-SpO_2_ (β, −0.294; 95% CI, −0.455 ~ −0.133; *p* = 0.001) and HLM score (β, 0.265; 95% CI, 0.088 ~ 0.412; *p* = 0.002) were independently correlated with PVR (Table 5).

**Table 4:** Univariate analysis between PVR and baseline characteristics

Characteristics	*t*	*ρ*	*P*-value
Age		0.230	0.020
Gender (male)	−1.035		0.305
Smoking history (smokers)	−1.279		0.207
BNP		−0.091	0.361
FVC, % pred		−0.124	0.211
D_L_CO,% pred		−0.518	< 0.001
PaO_2_		−0.424	< 0.001
PaCO_2_		−0.167	0.091
Six-minute walk distance		−0.583	< 0.001
Min-SpO_2_		−0.318	0.001
BALF-neutrophils, %		0.142	0.152
BALF-lymphocytes, %		0.098	0.325
BALF-eosinophils, %		0.113	0.255
HLM score		0.349	< 0.001

Definitions of abbreviations: *PVR* pulmonary vascular resistance, *t* Student’s *t*, *ρ* Spearman’s correlation coefficient, *BNP* brain natriuretic peptide, *FVC* forced vital capacity, *% pred* percent of normal predicted value, *D*_*L*_ *CO* diffusing capacity of the lung for carbon monoxide, *PaO*_*2*_ partial pressure of oxygen in arterial blood, *PaCO*_*2*_ partial pressure of carbon dioxide in arterial blood, *min-SpO*_*2*_ minimum oxygen saturation during 6-min walk test, *BALF* bronchoalveolar lavage fluid, *HLM* hemosiderin-laden macrophage

**Table 5:** Multivariate linear regression analysis for correlation with PVR

Characteristics	*β*	95% CI	*P*-value
Six-minute walk distance	−0.391	−0.587 ~ −0.196	< 0.001
min-SpO_2_	−0.294	−0.455 ~ −0.133	0.001
HLM score	0.265	0.088 ~ 0.412	0.002
			R = 0.595

Definitions of abbreviations: *PVR* pulmonary vascular resistance, *β* standardized partial regression coefficient, *95% CI* 95% confidence interval, *min-SpO*_*2*_ minimum oxygen saturation during 6-min walk test, *HLM* hemosiderin-laden macrophage

## Discussion

This is the first report to show a significant correlation between pulmonary hemodynamic parameters measured by RHC and HLM in BALF in patients with IPF. HLM score in particular was independently correlated with PVR, together with 6MWD and min-SpO_2_, which were evaluated by multivariate analysis.

Recently, several studies have reported that hemosiderin deposition or exaggerated numbers of HLM are observed in IPF [3, 4]. Puxeddu et al. reported elevated HLM in BALF of patients with IPF [4]. By comparing BALF data from 47 radiologically or histologically proven IPF patients with data from healthy controls, they found significantly higher HLM in the IPF group regardless of smoking history, which is compatible with our findings.

HLM accumulation in IPF has been conjectured to be related to pulmonary hemodynamics in association with several pathological findings, including increased alveolar septal capillary density and microvessel density [3, 25]. In the lung tissues of IPF patients, increased capillary density and angiogenesis were reported to exist in architecturally preserved lung areas next to the fibrotic areas [26–28]. Colombat et al. reported that the occlusion of venules and small pulmonary veins were observed in those architecturally preserved lung tissues in lung specimens from end-stage IPF cases, which were associated with hemosiderin deposition in the interstitium and alveolar macrophages [25]. These findings suggest that occult bleeding in the lungs of patients with IPF is caused by the vulnerability of abnormal blood vessels and elevated blood pressure due to occlusion of those blood vessels.

In 2010, Kim et al. demonstrated that iron deposition in nonfibrotic lung areas from IPF were significant predictors of RVSP measured by echocardiography [3]. In a recent study, Puxeddu et al. reported that IPF patients with higher RVSP (>35 mmHg) evaluated by echocardiography showed significantly elevated HLM compared to those with RVSP < 35 mmHg. In the present study, the HLM scores of patients with IPF were significantly correlated with pulmonary hemodynamic parameters. We also evaluated mPAP and PVR by RHC, and the correlation of PVR with HLM score was confirmed using multivariate analysis. 6MWD [11, 12] and min-SpO_2_ [9, 11] are well-known correlating factors for mPAP in patients with IPF. This study showed significant correlation between those factors and both mPAP (data not shown) and PVR, which is consistent with other reports on mPAP [9, 11, 12].

Although the specific BAL cellular pattern of IPF is unclear, BAL may be performed if IPF is suspected, especially if other suspected conditions need to be excluded for differential diagnoses [26, 29]. Evaluation of the HLM score, which might be correlated with PVR, can provide additional information from BALF of patients with IPF for the detection of PH in the early phase of the disease, and prompt clinicians to perform further evaluation for PH.

Several limitations of this study should be mentioned. First of all, this is a retrospective study in a single institute. Secondly, due to the high risk of performing BAL in patients with severe IPF, our cohort included patients with mainly mild to moderate IPF, and we cannot affirm that the results would be the same in all patients with IPF including severe cases. Nevertheless, our cohort showed a significant positive correlation between HLM score and PVR or mPAP. This fact suggests that hemosiderin deposition in the lungs of IPF is already significant even in mild to moderate IPF, and HLM score can reflect such early microvascular abnormality. Finally, pathological findings related with hemosiderin deposition were not reviewed for this study. Further pathological validation is needed to confirm our findings.

## Conclusions

Among the data from patients with idiopathic pulmonary fibrosis, hemosiderin-laden macrophages in bronchoalveolar-lavage fluid were significantly correlated with pulmonary hemodynamic parameters evaluated by right heart catheterization, and were an independent correlating factor of pulmonary vascular resistance.

## Abbreviations

6MWD: 6-minute walk distance
95% CI: 95% confidence interval
BAL: Bronchoalveolar lavage
BALF: Bronchoalveolar lavage fluid
D_L_CO: Diffusing capacity of the lung for carbon monoxide
HLM: Hemosiderin-laden macrophages
IPF: Idiopathic pulmonary fibrosis
min-SpO_2_: Minimum oxygen saturation during 6-minute walk test
mPAP: mean pulmonary arterial pressure
PVR: Pulmonary vascular resistance
RHC: Right heart catheterization
RVSP: Right ventricular systolic pressure
UIP: Usual interstitial pneumonia
β: Standardized partial regression coefficient
